# Supplementary material for: Infectious complications related to radiofrequency ablation of liver tumors: The role of antibiotics
Source: PLoS One. 2021 Nov 19;16(11):e0259641. doi: 10.1371/journal.pone.0259641 (PMC8604344; doi:10.1371/journal.pone.0259641)
Supplement: S1 File — (DOCX) [file pone.0259641.s001.docx]

**An exploratory clinical trial of prophylactic antimicrobial administration once before treatment in percutaneous radiofrequency ablation of hepatic malignancies**

# Table of Contents

1. Background
2. Purpose and Necessity
3. Overview of the test drug
4. Patients
5. Method of obtaining informed consent
6. Method
7. Evaluation Items
8. Observation and test items
9. Discontinuance criteria
10. Handling in case of adverse events
11. Handling of deviations from the implementation plan
12. Termination, suspension, or discontinuation of the study
13. Trial period
14. Data aggregation and statistical analysis methods
15. The target number of cases and rationale for setting
16. Consideration for human rights and safety and disadvantage of subjects
17. Compensation and insurance coverage for health problems
18. Compliance with GCP and Declaration of Helsinki
19. Record keeping
20. Research Funding and Conflicts of Interest
21. Registration of research plan and publication of research results
22. Research Organization
23. Changes to the implementation plan, etc.
24. Reference materials and bibliography

# Title

Independent clinical trial

"An exploratory clinical trial of prophylactic antimicrobial administration once before treatment in percutaneous radiofrequency ablation of hepatic malignancies."

# 1. Background

Percutaneous radiofrequency ablation of hepatic malignancies is widely performed in Japan as a less invasive treatment method compared to surgery. However, complications have been observed, and the incidence of serious complications (requiring treatment) was 2.8% among 5072 cases performed at the Department of Gastroenterology, the University of Tokyo, until December 2008.

Although percutaneous radiofrequency ablation is a hematological procedure, the wound is small and the risk of infection from the wound is very small. The mechanism of liver abscess is secondary infection of coagulated necrotic tissue, most likely due to transbiliary retrograde infection of bacteria in the duodenum, but also bacteremia due to percutaneous electrode needle puncture and possible perforation of the liver due to thermal injury of the adjacent gastrointestinal tract.

In all cases, antimicrobial therapy has been started immediately before treatment to prevent the development of liver abscesses and continued until the maximum daily body temperature falls below 37.5°C (minimum of 2 days, the day of treatment and the next day). Antimicrobial agents were mainly cefmetazole sodium before 2004, and flumoxef sodium after 2005. According to a questionnaire survey by the Japan Society of Hepatology and other organizations, prophylactic administration similar to that of our department is used in many institutions in Japan.

In other countries, prophylactic administration of antimicrobial agents is not usually used. Still, an increasing number of facilities are introducing prophylactic administration of antimicrobial agents (e.g., amoxicillin + clavulanic acid) once before treatment. However, its efficacy is unknown. The incidence of liver abscess reported from overseas is not clearly higher than that in our department or in Japan. There is a report that antimicrobial agents are administered for 2 to 5 days in high-risk cases of abscess, such as after cholangiopancreatic anastomosis, but there is no report of a method of administration in which antimicrobial agents are used every day until the fever is relieved in all cases, as in our department and other institutions in Japan. In Japan, including our department, radiofrequency ablation is generally not indicated for patients who have undergone surgery or papillotomy of the common bile duct.

Immediately after radiofrequency ablation, fever of 38°C or higher is observed in more than half of the patients, mostly due to thermal coagulation and necrosis of tissues, and it spontaneously resolves in a few days. Fever may also be caused by non-infectious complications such as hepatic infarction, atelectasis, or hematoma and may persist for up to a week. Conventionally, antimicrobial agents have been continued until the fever is relieved, but in these cases, antimicrobial agents may be considered unnecessary. On the other hand, fever that lasts for more than a week after treatment, or fever that appears after a period of time after the patient has recovered, should be suspected to be due to liver abscess. In fact, of the 16 cases of liver abscess we experienced in our department, 7 were diagnosed during the same hospitalization as treatment. The remaining 9 cases were diagnosed with symptoms such as fever after discharge from the hospital after completion of antimicrobial therapy. 2 cases were diagnosed within 1 month, 4 cases within 6 months, and 3 cases after 6 months from the start of treatment. Most of these cases required percutaneous or transpapillary abscess drainage.

In 14 cases, the causative organism was identified by blood culture or pus culture. Of these, 11 were enterococci, 1 was Pseudomonas aeruginosa, and in 2 cases, candida was detected simultaneously. In these cases, continuous administration of antimicrobial agents may induce multidrug-resistant organisms, making treatment difficult when bacterial infections such as liver abscesses occur, and it may be advantageous to start antimicrobial administration after bacterial infections become apparent, even in cases that result in liver abscesses.

In view of the above, it was considered desirable to reduce the dose of antimicrobial agents administered during radiofrequency ablation as much as possible without compromising safety. Therefore, we decided to discontinue the routine administration of antimicrobial agents in the postoperative period and to administer them only once before treatment, taking into consideration the situation in other countries.

# 2. Purpose and Necessity

The purpose of this study is to clarify the safety of prophylactic antimicrobial administration in percutaneous radiofrequency ablation of hepatic malignancies, i.e., the incidence of liver abscesses and other bacterial infections, when sulbactam/ampicillin (Unacin S) 3 g intravenous infusion is administered only once immediately before treatment and post-treatment antimicrobial administration is discontinued. In other countries, prophylactic antibiotics for percutaneous radiofrequency ablation are generally administered as a single dose before surgery, and therefore, a single dose before surgery was adopted in this study as well.

If liver abscesses and other bacterial infections do not increase after treatment, continuous administration of antimicrobial agents after treatment can be abolished, which would be beneficial in terms of decreasing the occurrence of resistant bacteria, decreasing side effects such as allergies and enteritis caused by antimicrobial agents, and reducing costs and the workload of medical staff.

# 3. Overview of the test drug

Brand name: Unacin S Generic name: sulbactam/ampicillin

# 4. Patients

All patients undergoing percutaneous radiofrequency ablation for hepatic malignancies in the Department of Gastroenterology will be included. The type of tumor, age, and initial recurrence are not important. If the same patient is hospitalized multiple times during the study period for percutaneous radiofrequency ablation due to tumor recurrence, etc., each hospitalization will be included in this study.

The following patients should be excluded. 1) Patients who do not agree to participate in the study. 2) Patients under 20 years of age. 3) Patients who have a history of allergy or other adverse reactions to the antimicrobial agents used in this study. 4) Patients who are judged inappropriate to participate in the study by the principal investigator and sub-investigators.

# 5. Method of obtaining informed consent

For this study, a consent explanation document approved by the Clinical Research Support Center of the University of Tokyo Hospital will be given to the patient, sufficient written and oral explanations will be given, and the patient's free and voluntary consent will be obtained in writing.

When information on efficacy or safety that may affect patient consent is obtained, or when changes are made to the implementation plan that may affect patient consent, the information will be promptly provided to the patient, and the patient's will regarding whether or not to participate in the study will be confirmed in advance.

# 6. Method

6.1. Study type and design

This is an uncontrolled exploratory clinical trial.

6.2. Outline of the Study

1) Flow of percutaneous radiofrequency ablation for hepatic malignancies

The procedure for percutaneous radiofrequency ablation is the same as before. On the day of admission, blood tests, chest and abdominal X-rays, and abdominal ultrasonography are performed. The treating physician will explain the treatment to the patient's family and obtain written consent. At that time, the study will be fully explained to the patient, and written consent for participation will be obtained.

Treatment is performed on the next business day after admission. Contrast-enhanced CT is performed on the next business day after the treatment to determine the effect of the treatment. If complete necrosis of the tumor is confirmed, the treatment is considered complete. Patients are discharged from the hospital if they have a daily maximum body temperature below 37.5°C, good food intake, and no symptoms such as abdominal pain. On the other hand, if a residual tumor is suspected by CT scan, additional treatment will be given after a minimum interval of 2 days. However, if a fever of 38.0°C or higher, ascites, pleural effusion, or deterioration of liver function is observed, treatment should be postponed until the patient recovers sufficiently. The follow-up after additional treatment is the same as after the first treatment. Patients will be discharged from the hospital if their maximum body temperature is below 37.5°C per day, food intake is good, and there are no symptoms such as abdominal pain. However, a CT scan to determine the efficacy of the treatment will be performed as an outpatient after discharge, and the patient will be readmitted if additional treatment is necessary.

2) Antimicrobial administration

Patients participating in this study will receive intravenous sulbactam/ampicillin (Unacin S) 3g prior to percutaneous radiofrequency ablation (upon entry to the treatment room). Thereafter, no antimicrobial agents will be administered until a liver abscess, or another bacterial infection is diagnosed.

3) Post-treatment examination and follow-up

After four hours of treatment, the treating physician will perform a physical examination. On the morning of the next day of treatment, a physical examination, general blood tests, and biochemical tests are performed. A contrast-enhanced CT scan will be performed in the afternoon of the day after treatment (or the following Monday if treatment is on a Friday).

After percutaneous radiofrequency ablation, fever of 38°C or higher is often observed even in uncomplicated patients and can also be caused by non-infectious complications such as liver infarction or atelectasis. Therefore, fever alone cannot be used to diagnose liver abscess or other infectious diseases. Even in the case of fever, if physical examination and blood tests do not reveal any symptoms suggestive of bacterial infection, the patient should be observed, and no antimicrobial agents should be administered.

If additional radiofrequency ablation is performed, intravenous infusion of Unacin S 3g is administered immediately before the treatment as well, and the details of subsequent follow-up are the same as for the first treatment. However, a contrast-enhanced CT scan is not performed in principle.

After discharge from the hospital, outpatient visits are scheduled within one month, and blood tests are performed. Blood tests and contrast-enhanced CT will be performed in 3-4 months. After that, patients will be followed up with CT and blood tests every 3-4 months in principle.

4) Treatment plan for liver abscess and other bacterial infections, etc.

If fever with cold shivering or more than 1°C above the previous day's maximum body temperature is observed, there is a possibility of complications from bacterial infection. If liver abscess is suspected, perform blood tests, blood cultures, ultrasonography, and contrast-enhanced CT scan if necessary. If a bacterial infection of other organs is suspected, a chest X-ray and urinalysis should also be performed.

If the above tests confirm the diagnosis of liver abscess, or if there is a strong clinical suspicion even if there are no definitive findings, antimicrobial agents should be re-administered promptly. The first choice of antimicrobial agent to be used is tazobactam/piperacillin (Zosyn, 4.5 g every 8 hours by intravenous infusion), referring to "Guidelines for the Use of Antimicrobial Agents" edited by the Japanese Association for Infectious Diseases and the Japanese Society of Chemotherapy, but a final decision will be made after careful consideration of each case. Percutaneous or transpapillary drainage of the abscess should be performed as needed. If blood or pus cultures reveal the causative organism, antimicrobial agents should be changed if appropriate.

If the patient develops bacterial infections other than liver abscess, such as pneumonia, urinary tract infection, or idiopathic bacterial peritonitis, the study should be stopped, and antimicrobial therapy started. In addition, if complications such as perforation of the gastrointestinal tract or gallbladder are observed, which generally require antimicrobial administration even if bacterial infection is not necessarily present at the time of onset, the study should be stopped, and antimicrobial administration should be started. In all cases, the choice of antimicrobial agent should be based on the pathology.

6.3. Planned Duration of Patient's Participation in the Study

The follow-up period for this study is two months from the date of the start of percutaneous radiofrequency ablation. Although there is a possibility that abscesses may occur after the 2-month follow-up period, in principle, all patients who underwent percutaneous radiofrequency ablation are followed up at the outpatient clinic in our department. Abscesses diagnosed after the follow-up period will also be used in the analysis of the results whenever possible.

# 7. Evaluation Items

7.1. Evaluation items for safety and efficacy

Primary endpoint: incidence at 2 months after treatment

Secondary endpoints: causative organism, number of days to the diagnosis of abscess, incidence of infections other than liver abscess (pneumonia, urinary tract infection, etc.), number of days with persistent fever, number of days of hospitalization, costs

# 8. Observation and test items

8.1. Before treatment

・Patient characteristics: age, gender, pre-existing medical conditions, medications, and presence of comorbidities

・Vital signs: body temperature, blood pressure, pulse rate, and SpO2

・Details of the tumor: diameter, number, and location of hepatic malignancies

・Blood tests: white blood cell count, red blood cell count, hemoglobin level, platelet count, CRP, total bilirubin, albumin, AST, ALT, γ-GTP, ALP, BUN, creatinine, and prothrombin time

・Standard 12-lead electrocardiogram

・Chest and abdominal x-ray

・Abdominal ultrasonography.

8.2. Post-treatment follow-up period

8.2.1 On the day of percutaneous radiofrequency ablation and during the subsequent hospitalization

・Vital signs: body temperature, blood pressure, pulse rate, SpO2

・Presence of abdominal pain, nausea and vomiting

8.2.2 After percutaneous radiofrequency ablation

1) The day after treatment

・Blood tests: white blood cell count, CRP, total bilirubin, AST, ALT, γ-GTP, ALP, and albumin

・Contrast-enhanced CT (If treatment is on a Friday, it will be performed on the next business day. However, if any complication is strongly suspected, contrast CT should be performed even on holidays. If contrast CT is not possible due to allergy to iodine contrast medium or renal impairment, non-contrast CT will be performed.)

2) If fever of 38°C or higher persists but general condition is good, on the 4th day after treatment

・Blood tests: white blood cell count, CRP, total bilirubin, AST, ALT, γ-GTP, ALP, and albumin

3) In case of worsening of vital signs, fever of 38°C or higher with chills and shivering, fever of 1°C or higher than the previous day's maximum body temperature, abdominal pain and vomiting, or other deterioration of general condition, the following tests should be performed promptly.

・Blood culture

・Blood tests: white blood cell count, CRP, total bilirubin, AST, ALT, γ-GTP, ALP, and albumin

・Abdominal ultrasound

・Chest x-ray, urinalysis, contrast CT, etc. as needed.

# 9. Discontinuation criteria

9.1. Discontinuation Criteria

・If the participant declines to participate in the study or withdraws his/her consent.

・If antimicrobial administration is deemed necessary due to complications of bacterial infections other than liver abscess.

・In any other cases where the study investigator deems it appropriate to discontinue the study.

9.2. Support after discontinuation

If the patient requests withdrawal, prophylactic administration of antimicrobial agents will be performed according to the conventional method. If a bacterial infection occurs, provide the necessary optimal treatment as soon as possible.

# 10. Handling in case of adverse events

When an adverse event is recognized, the principal investigator or sub-investigator should immediately take appropriate measures and record the event in the medical record. If treatment for an adverse event becomes necessary, the patient will be informed.

When the investigator recognizes the occurrence of a serious or important adverse event, the investigator shall promptly report it to the Hospital Director (Clinical Trial Department). The report should be the first report (emergency report) and the second report (detailed report). The deadline for reporting shall be within 15 days.

1) Definition of serious adverse events

・Death or threat of death

・Prolongation of hospital stay by more than 28 days

・Disability or threat of disability

・Disease or abnormality of later generation or congenital

2) Definition of important adverse events

・Complications of bacterial infections requiring prolongation of hospital stay for more than 14 days

# 11. Handling of deviations from the implementation plan

Any changes or revisions to the protocol or consent documents must be approved in advance by the Clinical Research Support Center of the University of Tokyo Hospital.

The principal investigator or sub-investigator may deviate from or change the study protocol for unavoidable reasons, such as to avoid an emergency, before obtaining prior approval from the Clinical Research Support Center. In such cases, the principal investigator or sub-investigator shall promptly submit to the Clinical Research Support Center the details of the deviation or change, the reason for the deviation or change, and a draft of the revised study protocol, if necessary, and obtain the approval of the Clinical Research Support Center and the hospital director.

If there are any deviations from the study protocol, the principal investigator or sub-investigator shall record the deviations along with the reasons for them, and the principal investigator shall report them to the hospital director. The principal investigator will keep copies of these records.

# 12. Termination, suspension, or discontinuation of the study

12.1 Termination of the study

At the end of the study, the principal investigator will promptly submit a study termination report to the hospital director.

12.2 Suspension or discontinuation of the study

The investigator will consider whether or not to continue the study if any of the following apply.

・If significant information regarding the quality, safety, or efficacy of the antimicrobial agent used in this study is obtained.

・If the interim analysis shows a significant increase in the incidence of liver abscess compared to the previous study.

・If it is judged to be impossible to achieve the planned number of patients within the study period due to the difficulty in obtaining consent for study participation.

・If the Clinical Research Support Center recommends or instructs discontinuation of the study, or instructs changes to the implementation plan, etc., and it is judged difficult to accept the changes.

When a decision is made to discontinue or suspend a study, the decision shall be promptly reported to the hospital director in writing with the reasons for the decision.

# 13. Trial period

From November 1, 2010 to October 31, 2012

# 14. Data aggregation and statistical analysis methods

All information obtained will be consolidated and anonymized, and stored on the LAN Disk installed in the office of the Department of Gastroenterology, the University of Tokyo. Control charts will be stored by the principal investigator in a locked vault in the laboratory. The LAN Disk is accessible only from the computers in each laboratory of the Department of Gastroenterology through the hospital LAN, and each registered user has a unique password (8 random alphanumeric characters) to ensure security. In addition, the hard disk itself is encrypted, making it impossible to decrypt without a USB key. Data management will be done by the principal investigator. All data and control tables will be destroyed after the completion of the study.

The principal investigator shall keep documents related to the study (copies of application documents, notification documents from the hospital director, copies of various application forms and reports, lists of subject identification codes, copies of consent forms, case reports, etc., and other documents or records necessary to assure the reliability of the data, etc.) for five years after the completion of the study. After that, the materials related to this study will be destroyed by shredding.

# 15. Target number of cases and rationale for setting

The occurrence of liver abscesses in 5076 patients who received the conventional method is a very rare event (0.3%) and considering reports from overseas; it is unlikely that the new method will significantly increase the number of abscesses. Therefore, even if a non-inferiority study were to be conducted to compare the conventional method with the new method, it would require a huge number of cases (>10,000), which is unrealistic. Therefore, we decided to conduct a one-arm study using patients treated with the conventional method as the historical control. The indications for radiofrequency ablation in our department have remained unchanged since its introduction in 1999, and there seems to be little bias in the historical control and the background conditions of the patients in this study.

Considering overseas reports and other factors, it is considered reasonable to set the maximum allowable incidence of liver abscesses with the new administration method, which requires only one dose of antimicrobial agent before surgery, at around 1%. Therefore, in this study, we considered the risk-benefit and economic efficiency of the conventional method and the new method, and judged that the new method was useful if the true incidence of liver abscesses with the new method was less than 1%.

Assuming that the threshold incidence rate is 1%, the expected incidence rate of liver abscess in the study treatment is 0.3%, the significance level is 5% on one side, and the power is 80%, the required number of enrollment based on the binomial distribution is 751 cases, and the expected number of enrollment is 760 cases with some dropouts.

If liver abscesses occur in more than 8 cases at the end of follow-up of all enrolled patients, it cannot be concluded that this therapy is effective because the incidence of liver abscesses does not exceed 1%, and the conventional method will be used thereafter.

If the number of patients is 7 or less, it cannot be concluded that the new administration method significantly increases the incidence of abscesses, so the new administration method will be continued after the completion of the study, and further detailed analysis will be conducted to determine the appropriateness of adopting the new administration method.

# 16. Consideration for human rights and safety and disadvantage of subjects

16.1. Consideration for human rights and protection of personal information

When handling raw data and consent forms, etc., related to the implementation of the study, sufficient consideration will be given to the protection of the confidentiality of the patients. Case reports and other documents to be submitted outside the hospital will use subject identification codes. When the results of a study are made public, information that can identify the subject should not be included. The data of the patients obtained in the study were not to be used for any other purpose than that of the study.

16.2. Consideration for safety and adverse effects

In the event of an adverse event such as a liver abscess, appropriate medical care and treatment should be provided promptly.

16.3 Patient's cost burden

This is within the scope of normal insurance treatment, and there will be no new cost burden compared to the past.

# 17. Compensation and insurance coverage for health problems

Any health problems arising from this study will be treated by normal insurance and no special compensation will be provided to patients. The investigators and sub-investigators will be covered by liability insurance.

# 18. Compliance with GCP and Declaration of Helsinki

This study shall be conducted in accordance with GCP. In addition, it will be conducted in compliance with the Declaration of Helsinki (revised in 2008).

# 19. Record keeping

The principal investigator shall retain documents related to the implementation of the study, etc. (copies of application documents, notification documents from the hospital director, copies of various application forms and reports, copies of patient identification code lists, consent forms, case report forms, etc., and other documents or records necessary to ensure the reliability of the data), and dispose of them after a prescribed period (e.g., 5 years after the publication of the study).

# 20. Research Funding and Conflicts of Interest

This study will be conducted within the scope of insurance coverage, but only the purchase of study drugs will be funded by research funds. There are no "possible conflicts of interest" in the design, conduct, or reporting of this study that would affect the results of the study or the interpretation of the results.

# 21. Registration of research plan and publication of research results

Prior to the start of the study, the details of the study plan will be registered in the public registration system (University Hospital Medical Information Network: UMIN). The results obtained from this study will be presented by the principal investigator and the sub-investigators at domestic and international conferences and in English papers after the completion of the study in order to contribute to the development of medical science.

# 22. Research Organization

Contact：Department of Gastroenterology, the University of Tokyo

7-3-1, Hongo, Bunkyo-ku, Tokyo 113-8655, Japan

Tel: 03-3815-5411

FAX: 03-3814-0021

# 23. Changes to the implementation plan, etc.

Any changes (revisions) to the study protocol or consent documents must be approved in advance by the Clinical Research Support Center of each medical institution.

# Reference materials and bibliography

1. Kasugai H, Osaki Y, Oka H, Kudo M, Seki T. Severe complications of radiofrequency ablation therapy for hepatocellular carcinoma: an analysis of 3,891 ablations in 2,614 patients. Oncology 2007;72 Suppl 1:72-75.

2. de Baere T, Risse O, Kuoch V, Dromain C, Sengel C, Smayra T, Gamal El Din M, et al. Adverse events during radiofrequency treatment of 582 hepatic tumors. AJR Am J Roentgenol 2003;181:695-700.

3. Kong WT, Zhang WW, Qiu YD, Zhou T, Qiu JL, Zhang W, Ding YT. Major complications after radiofrequency ablation for liver tumors: analysis of 255 patients. World J Gastroenterol 2009;15:2651-2656.

4. Livraghi T, Meloni F. Treatment of hepatocellular carcinoma by percutaneous interventional methods. Hepatogastroenterology 2002;49:62-71.

5. Mulier S, Mulier P, Ni Y, Miao Y, Dupas B, Marchal G, De Wever I, et al. Complications of radiofrequency coagulation of liver tumours. Br J Surg 2002;89:1206-1222.

6. Rhim H. Complications of radiofrequency ablation in hepatocellular carcinoma. Abdom Imaging 2005;30:409-418.
