# Supplementary material for: Infectious complications related to radiofrequency ablation of liver tumors: The role of antibiotics
Source: PLoS One. 2021 Nov 19;16(11):e0259641. doi: 10.1371/journal.pone.0259641 (PMC8604344; doi:10.1371/journal.pone.0259641)
Supplement: Attachment — Submitted filename: Response to Reviwers PONE-D-21-03580R.docx [file pone.0259641.s003.docx]

Dear Dr. Jee-Fu Huang

Thank you very much for reviewing our manuscript. We are herewith sending you the revised manuscript. We are deeply appreciative of numerous helpful suggestions by the associate editor and reviewers. We believe that the paper is much improved with revision, and we hope that you will find it acceptable for publication.

**Point-by-Point Responses to Reviewers’ Comments**

Reviewer #1:

1. Between February 1, 1999 and November 30, 2010, a total of 6,763 RFA treatments were performed in 2,355 patients with liver malignancy and was categorized in the group of administered continuous prophylactic antibiotics. Since this article analyzed their data using RFA treatment times. It means that this historical control group will have higher recount rates (mean RFA treatment times will approach 3 for each patient) compared to the other groups (mean RFA treatment times less than 2 for each patient). Higher TACE experience and tumor size was noted in historical control group, Considering the higher recount rates in these patients when doing statistical analysis is needed to avoid mis-interpretation especially when trying to find any risk factors which contributed to later infection complication after RFA.

[Reply]

As the reviewer pointed out, the patient in the continuous administration group essentially had a longer follow-up period than the single administration group. In addition, since we analyzed data on a treatment basis, some patients who underwent RFA in both periods were included in both groups. Patients with a single administration of antibiotics may have an infectious complication related to the previous RFA performed with continuous administration of antibiotics. To avoid recounting, we associated cases with liver abscess with each group based on the cite of RFA.

According to the reviewer’s comment, we added the following sentences to the Methods.

Since some patients underwent RFA with single and continuous administration during their clinical course, to avoid recounting, we associated a treatment with liver abscess with each group based on the site of RFA.

2. There seemed to be a typo in page 7. study protocol: line 4, antibiotics until fever "redTuced " following RFA. Please correct it.

[Reply]

Thank you for your comment. As the reviewer pointed out, this is a typo. redTuced is corrected to reduced.

3. It seemed not very appropriate to include infection complication occurred 300 more days later after RFA treatment. To address antibiotic prophylaxis issue, included infection events within 30 days after RFA may be more appropriate and realistic.

[Reply]

We agree with the reviewer’s comments. However, the infectious complications that occurred several months later were potentially related to RFA when they occurred in the ablated area. To avoid underreporting, we included all cases with an infection found in a previously ablated site.

4. Previous biliary intervention history or if co-existed cholelithiasis; grade of CTP score or MELD score are not included in the analysis, these factors could also be confounding or contributing risk factors when doing comparison.

[Reply]

We appreciate this constructive comment. We regard those with a history of bilioenteric anastomosis or sphincterotomy as a contraindication of RFA. We added the following description to the Methods.

We also excluded patients with a history of bilioenteric anastomosis or sphincterotomy.

Unfortunately, our database does not include the status of ascites and encephalopathy at each RFA treatment except for the initial treatment. Instead, we included albumin and bilirubin, which are the component of CTP, in the analysis.

5. The data can't tell if antibiotic prophylaxis is really needed for HCC RFA. Authors may still should address this issue regarding the need of antibiotics prophylaxis for HCC RFA from literature review in discussion section.

[Reply]

As the reviewer pointed out, the administration of antibiotics during RFA may be unnecessary. We have already referred to this point in the first paragraph of the Discussion as follows.

…. antibiotic use may not be prophylactic at all, although this study cannot provide evidence for nonuse of prophylactic antibiotics.

We added the following statement.

Indeed, some institutions did not use antibiotics before RFA [14, 19, 20]. Bhatia et al. reported that infectious complications occurred in 1 out of 121 RFA sessions in which antibiotics were not administered.

Reviewer #2:

1. This study included metastatic liver tumors hence we are wondering how many patients were primary GI-tract malignancies that might predispose to post-RFA infections because 4 in 844 developed infection episodes compared to 20 out of 9,792 in the hepatocellular carcinoma (HCC) patients?

[Reply]

The breakdown of 844 cases of metastatic liver tumor is as follows: colorectal cancer in 547, breast cancer in 70, gastric cancer cases in 66, gastrointestinal stromal tumor in 38, neuroendocrine tumor in 32, pancreatic ductal cancer in 14, esophageal cancer cases in 12, and others in 65. Most of the primary sites are GI tracts. We consider those with metastatic tumors may have a higher risk of infectious complications because of larger tumor size than HCC. Since we excluded patients with a history of enterobiliary anastomosis from the indication of RFA, we do not regard GI-tract cancer itself as a risk factor.

2. Some patients received TACE before RFA, please address their baseline tumor staging in the context.

[Reply]

We appreciate this comment. Since we regard TACE + RFA as a combination therapy to enhance the effect of RFA, the baseline tumor staging was on the condition before TACE. We added the following sentence to the Methods.

In cases with TACE plus RFA combination therapy, we adopted data regarding tumor characteristics before TACE.

3. Since the number of tumor nodules was not a risk factor for an infectious complication after RFA by univariate analysis, I recommend the authors clarifying that why ‘the number of ablation sessions’ and ‘the duration of ablation’ were not the risk factors for post-RFA infectious complications.

[Reply]

Thank you for your comment. Theoretically, a larger number of sessions and longer ablation time should be associated with a large ablation area. However, the number of events may be too small to detect a statistically significant difference.

We added the following sentence to the Discussion.

The number of nodules was not associated with an increased risk. Theoretically, the risk increases according to the number of ablated sites. However, the small number of events may be too small to detect a statistically significant difference.

4. Will the liver fibrosis stage influence the infectious complications after RFA (e.g. using FIB-4, Child-Pugh, APRI, or MELD scores instead of albumin, T. bil., AST, ALT, and platelet count)?

[Reply]

We appreciate this constructive comment. Unfortunately, our database does not include the status of ascites and encephalopathy at each RFA treatment except for the initial treatment. Instead, we included albumin and bilirubin, which are the component of CTP, in the analysis. The serum creatinine levels are also not available in early cases.

We added the FIB-4 index and APRI score in the analysis.

5. Since the post-RFA infectious complications were acceptably low under both single or multiple prophylactic antibiotics and the maximal tumor diameter was the only risk factor, will the authors recommend prophylactic antibiotics applied only to the selected patients?

[Reply]

As the reviewer pointed out, the administration of antibiotics during RFA may be unnecessary. We have already referred to this point in the first paragraph of the Discussion as follows.

…. antibiotic use may not be prophylactic at all, although this study cannot provide evidence for nonuse of prophylactic antibiotics.

We also added the following statement.

Indeed, some institutions did not use antibiotics at all before RFA [14, 19, 20]. Bhatia et al. reported that infectious complications occurred in 1 out of 121 RFA sessions in which antibiotics were not administered.

6. Typo at line 4, 1st paragraph, PATIENT AND METHODS, Study protocol, ‘redTuced’, please correct it.

[Reply]

As the reviewer pointed out, this is a typo. redTuced is corrected to reduced.
