# Supplementary material for: Infectious complications related to radiofrequency ablation of liver tumors: The role of antibiotics
Source: PLoS One. 2021 Nov 19;16(11):e0259641. doi: 10.1371/journal.pone.0259641 (PMC8604344; doi:10.1371/journal.pone.0259641)
Supplement: Attachment — Submitted filename: Response to Reviwers PONE-D-21-03580R3.docx [file pone.0259641.s004.docx]

Dear Dr. Jee-Fu Huang

Thank you very much for reviewing our manuscript. We are herewith sending you the revised manuscript. We are deeply appreciative of numerous helpful suggestions by the associate editor and reviewers. We believe that the paper is much improved with revision, and we hope that you will find it acceptable for publication.

**Point-by-Point Responses to Reviewers’ Comments**

Reviewer #3:

1. If one patient was included in both groups for analysis, a hierarchical modeling technique may be used to account for within-subject correlation. Further, details should be added (e.g . n and % of patients included in both groups. A sensitivity analysis may be considered for a clean dataset with the exclusion of double counted patients.

[Reply]

We appreciate the reviewer’s constructive comment. We applied a linear mixed model to the data by using “lme4” package of the R considering the individual effect. The regression coefficients are almost similar to the standard logistic regression by the analysis.

During the entire study period, the number of individual patients was 3490. The breakdown was 1951 in the continuous administration group only, 856 in the single administration group only, and 683 patients in both groups. Excluding the 683 patients who overlapped in both groups, the total number of treatments was 8661, and the number of infectious complications was 18. We also performed a sensitivity analysis using a dataset without overlap. The results are as follows.

We added the following sentences and Table 4 to the Results.

RESULTS

As a total, 3490 individual patients were included in the current study; 1951 were solely in the continuous administration group, 856 were solely in the single administration group, and 683 were in both groups.

An ad-hoc analysis with a dataset excluding overlap cases showed similar results (Table 4).

# Table 4. An ad-hoc analysis with a dataset excluding overlap cases. Univariable and multivariable analyses of infectious complications.

|  | Univariable | | Multivariable | |
| --- | --- | --- | --- | --- |
| Variable | OR (95% CI) | P-value | OR (95% CI) | P-value |
| Age per year | 1.03 (0.98–1.09) | 0.27 |  |  |
| Age > 63 years | 2.67 (0.52–9.87) | 0.28 |  |  |
| Male gender | 0.74 (0.26–2.08) | 0.57 |  |  |
| HCC vs non-HCC | 0.66 (0.15–2.89) | 0.58 |  |  |
| Prior TACE | 3.16 (1.18–8.44) | 0.02 | 2.11 (0.75–5.88) | 0.16 |
| Maximal tumor diameter (mm) | 1.05 (1.03–1.07) | <0.001 | 1.05 (1.03–1.07) | <0.001 |
| Number of nodules | 1.13 (0.92–1.39) | 0.24 |  |  |
| Serum albumin | 0.66 (0.27–1.60) | 0.36 |  |  |
| Total bilirubin > 0.9 mg/dL | 1.41 (0.56–3.54) | 0.47 |  |  |
| AST > 45 U/L | 0.65 (0.25–1.67) | 0.37 |  |  |
| ALT > 35 U/L | 1.02 (0.41–2.58) | 0.96 |  |  |
| Platelet count > 11.2 × 10^4^/mm^3^ | 2.73 (0.97–7.68) | 0.06 |  |  |
| DM | 1.97 (0.76–5.09) | 0.16 |  |  |
| FIB-4 Index | 0.93 (0.81–1.07) | 0.32 |  |  |
| APRI score | 0.85 (0.56–1.31) | 0.47 |  |  |
| Single vs. continuous administration of antibiotics | 0.73 (0.26–2.05) | 0.55 | 1.29 (0.43–3.88) | 0.66 |

Abbreviations: ALT, alanine transaminase; APRI, aspartate aminotransferase to platelet ratio index; AST, aspartate aminotransferase; CI, confidence interval; DM, diabetes mellitus; FIB-4, Fibrosis-4; HCC, hepatocellular carcinoma; OR, odds ratio; TACE, transcatheter arterial chemoembolization; U/L, unit/liter.

2. Perhaps it is fair to add a comparison between N=348 vs N=766 during the overlapping time period.

[Reply]

We have checked the number of infectious complications in N=348 and N=766. Infectious complications occurred in 0 and 4 patients, respectively. The Fisher’s exact test does not show statistical significance (P = 0.32). Since we do not think the results provide additional information because of the small sample size, we did not add this analysis to the mamuscript

3. Add methods used for comparisons in Table 1 to Statistical Analysis

[Reply]

We appreciate this constructive comment. We added the following description to the Table 1 legends.

* Comparisons were made between the continuous and single administration protocols using the t-test.

4. Add upper limit to all reported infection complication rates

[Reply]

We appreciate this constructive comment. We added the following description to the Methods, with a 95% confidence interval for each infectious complication rate.

Confidence intervals (CI) for the rate of infectious complications were calculated based on a binomial distribution.

5. Use “univariable and multivariable” instead of “univariate and multivariate” throughout the text.

[Reply]

According to the reviewer’s comments, we have corrected "univariate" and "multivariate" to "univariable" and "multivariable" in the text.
